# Supplementary material for: Toward Pathogenic Biofilm Suppressors: Synthesis of Amino Derivatives of Pillar[5]arene and Supramolecular Assembly with DNA
Source: Pharmaceutics. 2023 Jan 31;15(2):476. doi: 10.3390/pharmaceutics15020476 (PMC9966598; doi:10.3390/pharmaceutics15020476)
Supplement: Supplementary file 1 [file pharmaceutics-15-00476-s001.zip › pharmaceutics-2151972-supplementary.pdf]

Toward Pathogenic Biofilm Suppressors: Synthesis of Amino Derivatives of Pillar[5]arene and Supramolecular Assembly with DNA

Yulia I. Aleksandrova [1], Dmitriy N. Shurpik [1],* Viktoriya A. Nazmutdinova [1], Olga A. Mostovaya [1], Evgeniya V. Subakaeva [2], Evgeniya A. Sokolova [2], Pavel V. Zelenikhin [2], Ivan I. Stoikov [1,]*

[1] Kazan Federal University, A.M. Butlerov Chemistry Institute, 420008 Kremlevskaya, 18, Kazan, Russian Federation
[2] Kazan Federal University, Institute of Fundamental Medicine and Biology, 420008 Kremlevskaya, 18, Kazan, Russian Federation

E-mail: Ivan.Stoikov@mail.ru; Fax: +7-8432-752253; Tel: +7-8432-337463

Electronic Supplementary Information (20 pages)

1. Materials and methods.........2

2. NMR, MALDI TOF MS, IR spectra of the compounds 3-6.........4

3. Dynamic light scattering.........14

4. UV-vis study.........16

5. Biological research.........20

8. References.........20

1. Materials and methods

Pillar[5]arene **1** were synthesized according to the literature procedure S1, pillar[5]arene **2-**literature procedure S2

Most chemicals were purchased from Aldrich and used as received without additional purification. Organic solvents were purified in accordance with standard procedures.

^{1}H NMR, ^{13}C NMR spectra were obtained on a Bruker Avance-400 spectrometer ($^{13}C\{^{1}H\}$ – 100 MHz and ^{1}H – 400 MHz). Chemical shifts were determined against the signals of residual protons of deuterated solvent (DMSO-d_6, $CDCl_3$). The concentration of sample solutions was 3–5%.

Attenuated total internal reflectance IR spectra were recorded with Spectrum 400 (Perkin Elmer) Fourier spectrometer. The IR spectra from 4000 to 400 cm^{-1} were considered in this analysis. The spectra were measured with 1 cm^{-1} resolution and 64 scans co-addition.

Elemental analysis was performed with Perkin Elmer 2400 Series II instrument.

Mass spectra (MALDI-TOF) were recorded on an Ultraflex III mass spectrometer in a 4-nitroaniline matrix.

Melting points were determined using a Boetius Block apparatus.

Additional control of the purity of compounds and monitoring of the reaction were carried out by thin-layer chromatography using Silica G, 200 µm plates, UV 254.

Fluorescence spectra were recorded on a Fluorolog 3 luminescent spectrometer (Horiba Jobin Yvon). The excitation wavelength was selected as 290 nm. The emission scan range was 300–550 nm. Excitation and emission slits were 5 nm. Quartz cuvettes with optical path length of 1 cm were used. The cuvette was placed at the front face position to avoid the inner filter effect. Fluorescence spectra were automatically corrected by the Fluoressence program. The spectra were recorded in 50 mM Tris HCl buffer (pH =6.5) with concentration of pillar[5]arenes 10 µM. For the mixture of pillar[5]arene **4-6** with DNA, the following concentrations were used: 10 µM macrocycle 4 and 60 µM DNA. The obtained molar ratio of pillar[5]arene **4-6** to salmon sperm DNA was 1:6. The experiment was carried out at 293 K.

UV–*vis* spectra were recorded using the Shimadzu UV-3600 spectrometer; the cell thickness was 1 cm, slit width 1 nm. Deionized water with a resistivity >18.0 MΩ cm was used to prepare the buffers (pH = 6.5). Deionized water was obtained from a Millipore-Q purification system. The absorption spectra of the mixtures of compounds **4-6** (1×10^{-5} M) with salmon sperm DNA were recorded after mixing the solutions at 298 K. The 6×10^{-4} M base pairs solution of the salmon sperm DNA (100, 150, 300, 450, 600, 750, 1200 6×10^{-4} M base pairs) in phosphate buffer was added to 300 µl of the solution of **4-6** (1×10^{-4} M) in 50 mM Tris HCl and diluted to final volume of 3 ml with buffer. The UV spectra of the solutions were then recorded. The results were calculated using the hyperchromic effect at a wavelength of 250 nm. The association constants of complexes were calculated by BindFit. Three independent experiments were carried out for each series. Student's t-test was applied in statistical data processing. The experiment was carried out according to the literature method.

Dynamic light scattering (DLS). The particle size and zeta potential were determined by the Zetasizer Nano ZS instrument at 20 ºC. The instrument contains 4mW He-Ne laser operating at a wave length of 633 nm and incorporated noninvasive backscatter optics (NIBS). The measurements were performed at the detection angle of 173° and the software automatically determined the measurement position within the quartz cuvette. The 1×10^{-4}–1×10^{-6} M of the **4-6**, salmon sperm DNA, **4-6** with salmon sperm DNA complex (concentration ratio 2:1, 1:1, 1:2, 1:3, 1:4, 1:5, 1:6 и 1:10) in 50 mM Tris HCl buffer (pH = 6.5) were prepared. Electrophoretic mobility of different samples was using a fold capillary cuvette (Folded Capillary Cell DTS1060, Malvern, U.K.). The experiments were carried out for each solution in triplicate.

Transmission electron microscopy (TEM). TEM analysis of samples was carried out using the JEOL JEM 100CX II transmission electron microscope. For sample preparation, 10 μl of the suspension 10^{-5} M were placed on the Formvar/carbon coated 3 mm nickel grid, which was then dried at room temperature. After complete drying, the grid was placed into the transmission electron microscope using special holder for microanalysis.

Biological experiments. Cytotoxicity. The ability of macrocyclic compounds **4** to inhibit the viability and proliferative activity of LEC cells and A549 cells was investigated using the MTT test according to S3. A549 – human alveolar adenocarcinoma cell line (ATCC, Rockville, Maryland, USA) and cow embryo lung epithelial (LEC) cells (Animal Cell Culture Collection of Russian Academy of Agricultural Science, Kazan, Russia) were cultured in DMEM medium supplemented with 10% serum and 100 Units/ml of penicillin and streptomycin in a humid atmosphere with 5% CO_2 at 37 ºC. Cells were seeded in 96-well plates at a concentration of 10^4 cells/well. After 24 h of cultivation, the medium was removed from the wells and replaced with a fresh one with the addition of the test substances. The volume of the culture medium in the wells was 100 μl. After 24 h (in experiments with pillararenes 4) incubation of cells in the presence of substances, the medium in the wells was replaced with a fresh one containing MTT reagent at a final concentration of 0.5 mg/ml. The cells were incubated with MTT for 4 h at 37 ºC in an atmosphere of 5% CO_2. Then, the medium was aspirated from the wells and 100 μl of dimethyl sulfoxide was added thereto, after which it was incubated at 37 ˚C for 15 min in the dark until the formazan crystals dissolve. The optical density of the formazan solution in the wells was measured using a plate reader (BioRad xMarkTM Microplate Spectrophotometer, USA) at a wavelength of 570 nm. Three series of experiments were carried out with 5 replicates for each variant in the series.

Formation of *St. aureus* biofilms. Preparing procedures: we made different concentrations of **4** pillararene ($C_{initial}=1\times10^{-3}$ M), diluted dist. Water (dilution series 1×10^{-3} M, 1×10^{-4} M, 1×10^{-5} M, 1×10^{-6} M). 100 μl of pillararene solutions were applied to the 24-well culture plate wells and dried for 48 h.

Culture of *Staphylococcus aureus* were grown in L-broth for 18 h to a density of 1.5×10^{11} cells/ml. Then, the culture was diluted in 20 times and 800 μl were added to the pillararene solutions treated wells. Samples were cultivated at 37°C for 48 hours until the formation of stable biofilms. Biofilms formed after 48 hours of cultivation were washed with sodium phosphate buffer (pH = 7.2) from planktonic cells, dried under sterile conditions during 24 hours , and stained with 0.1% gentian violet solution for 20 minutes. Stained biofilms were washed three times with sodium phosphate buffer (pH = 7.2) and dried. The thickness of the biofilm was determined by extraction with 96% ethanol the gentian violet dye bound to the biofilm matrix. Light absorption measurements in eluate samples were measured at λ= 570 nm on a BIO-Rad xMark Microplate spectrophotometer.

The average values for the data groups and the confidence interval of the populations ($p\leq0.05$) are calculated. Significance of group data differences were calculated in the variants without surface modification using the nonparametric Mann-Whitney test. The data were given in relative units. In the calculations, the light absorption of the eluate was taken as a unit in the variants without surface modification.

2. NMR, MALDI TOF MS, IR spectra of the compounds 3-6

Figure S1. ^{1}H NMR spectrum of 4,8,14,18,23,26,28,31,32,35–deca-((hydrazidocarbonil-2-sulfanedyl)ethoxy)-pillar[5]arene **(3)**. DMSO-d_6, 298 K, 400 MHz.

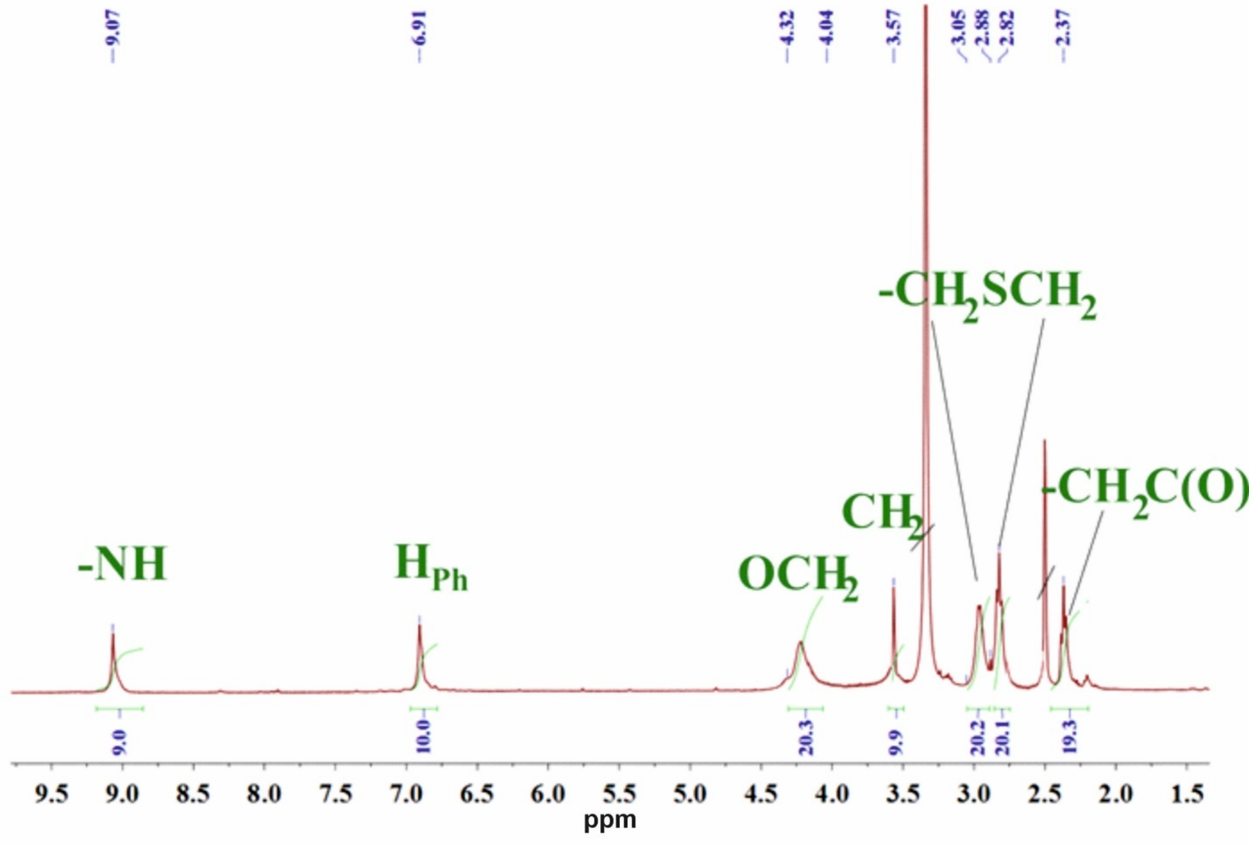

Figure S2. ^{1}H NMR spectrum of 4,8,14,18,23,26,28,31,32,35–deca-((2-(N,N-diethyl)ethylcarbamoyl-2-sulfanedil)ethoxy)-pillar[5]arene **(4)**. DMSO-d_6, 298 K, 400 MHz.

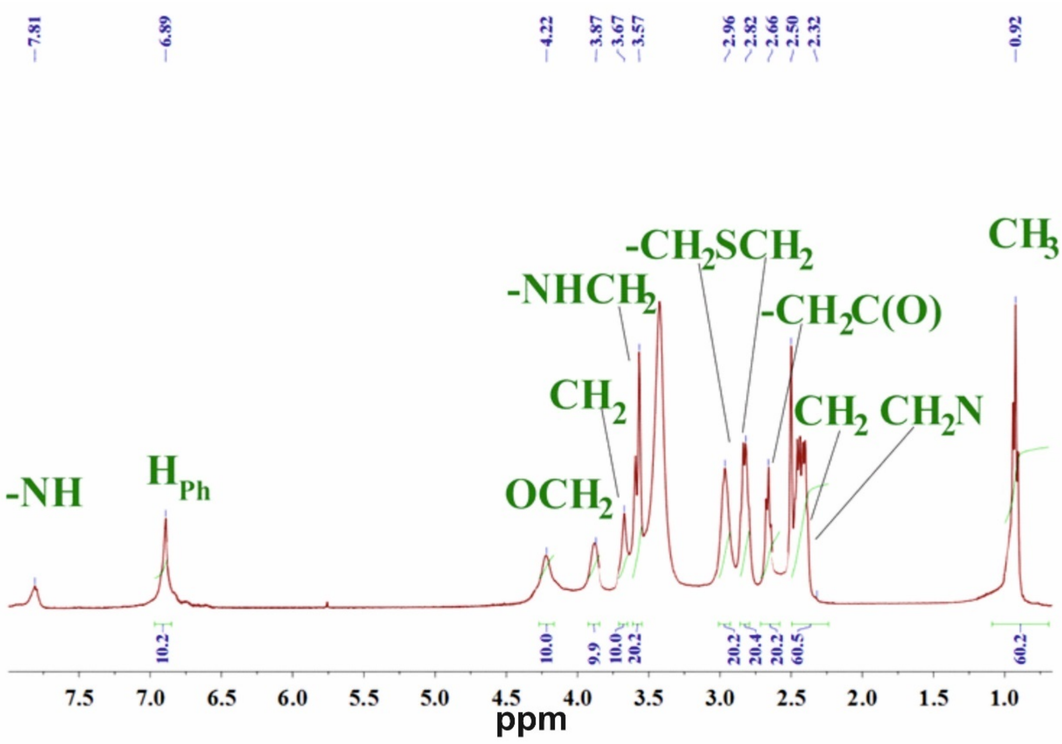

Figure S3. ^{1}H NMR spectrum of 4,8,14,18,23,26,28,31,32,35-deca-((2-(N,N-dimethyl)ethylcarbamoyl-2-sulfanedil)ethoxy)-pillar[5]arene **(5)**. DMSO-d_6, 298 K, 400 MHz.

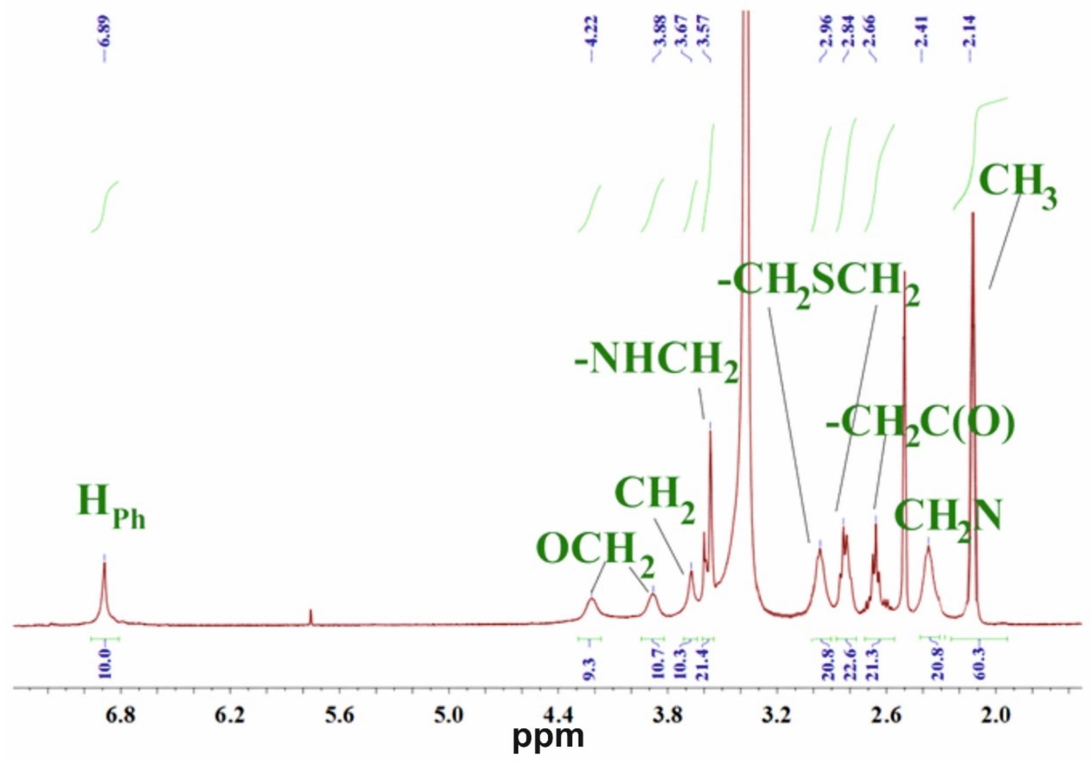

Figure S4. ^{1}H NMR spectrum of 4,8,14,18,23,26,28,31,32,35-deca-((2-(morfolin)ethylcarbamoyl-2-sulfanedil)ethoxy)-pillar[5]arene **(6)**. DMSO-d_6, 298 K, 400 MHz.

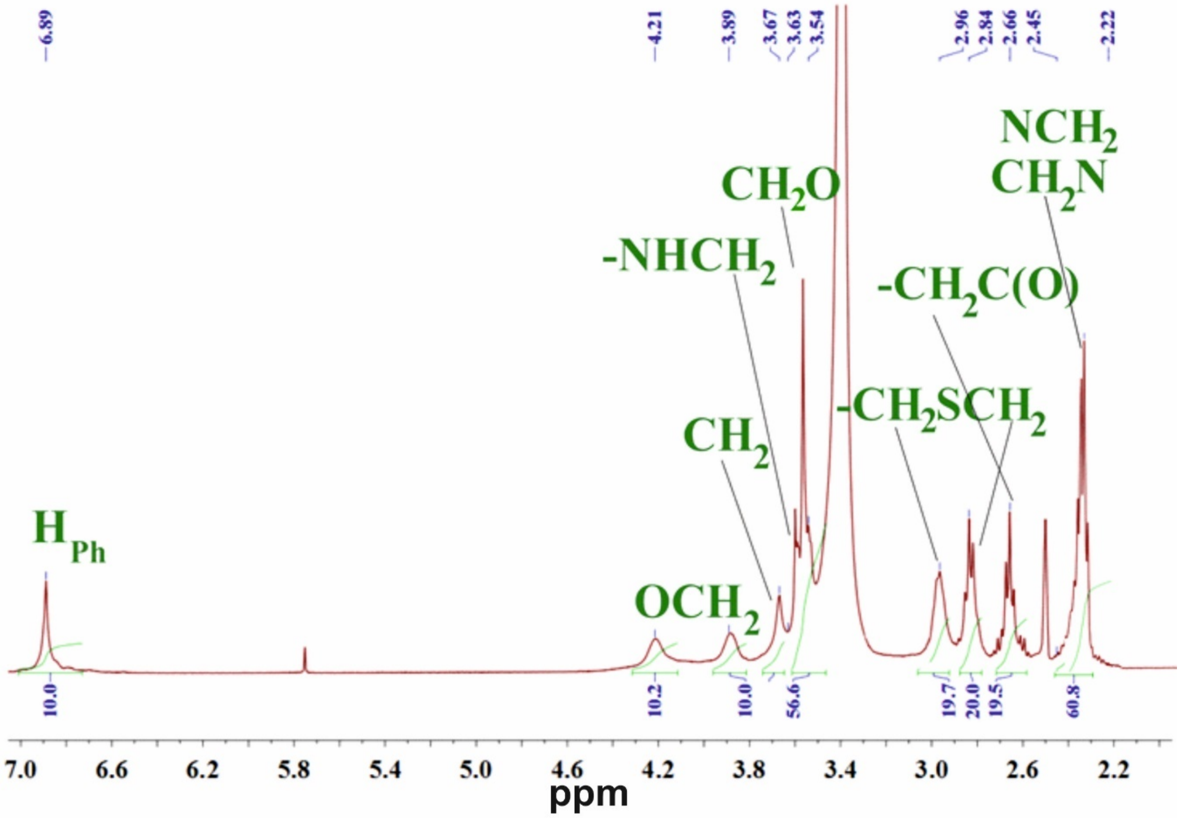

Figure S5. ^{13}C NMR spectrum of 4,8,14,18,23,26,28,31,32,35–deca-((hydrazidocarbonil-2-sulfanedyl)ethoxy)-pillar[5]arene **(3)**. DMSO-d_6 298 K, 400 MHz.

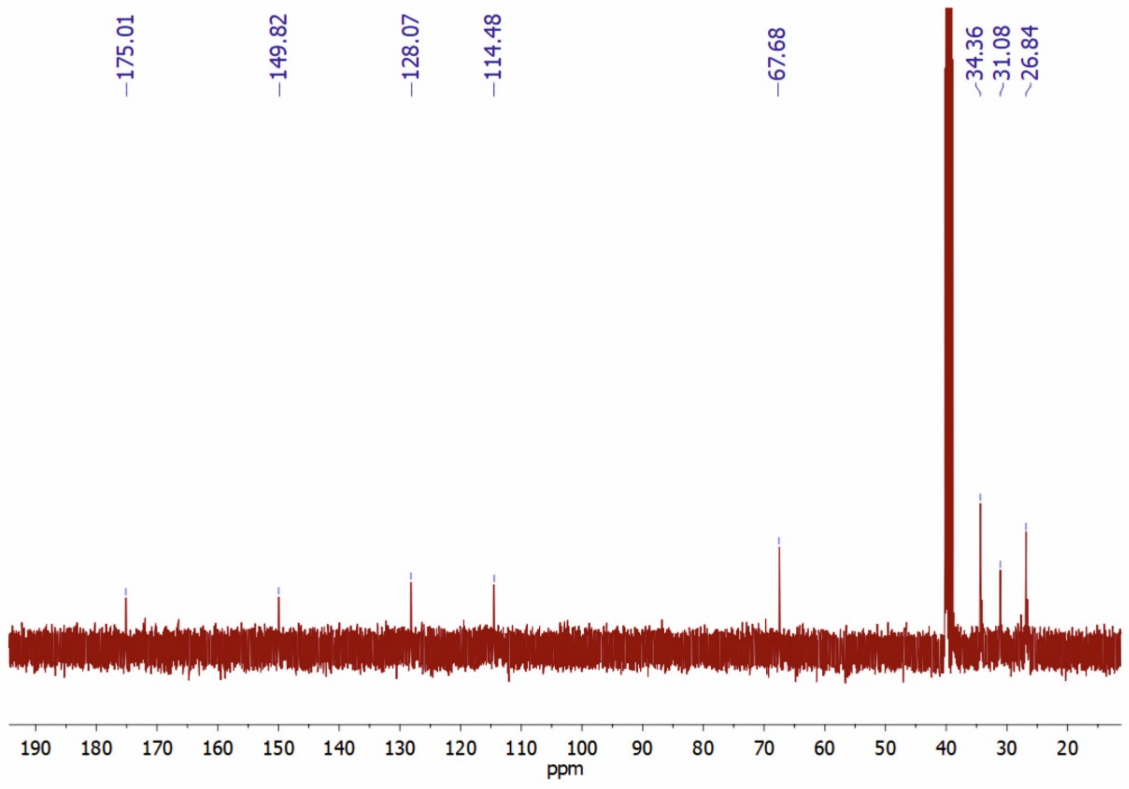

Figure S6. ^{13}C NMR spectrum of 4,8,14,18,23,26,28,31,32,35–deca-((2-(N,N-diethyl)ethylcarbamoyl-2-sulfanedil)ethoxy)-pillar[5]arene **(4)**. DMSO-d_6, 298 K, 400 MHz.

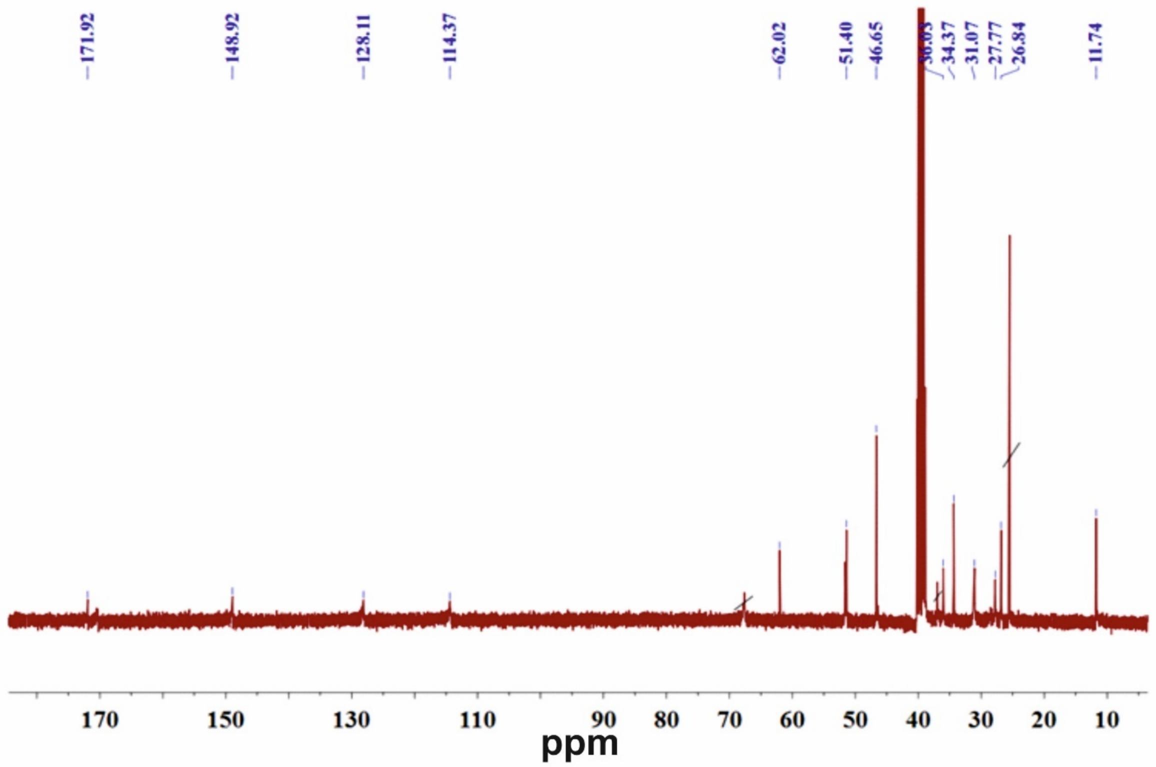

Figure S7. ^{13}C NMR spectrum of 4,8,14,18,23,26,28,31,32,35-deca-((2-(N,N-dimethyl)ethylcarbamoyl-2-sulfanedil)ethoxy)-pillar[5]arene **(5)**. DMSO-d_6, 298 K, 400 MHz.

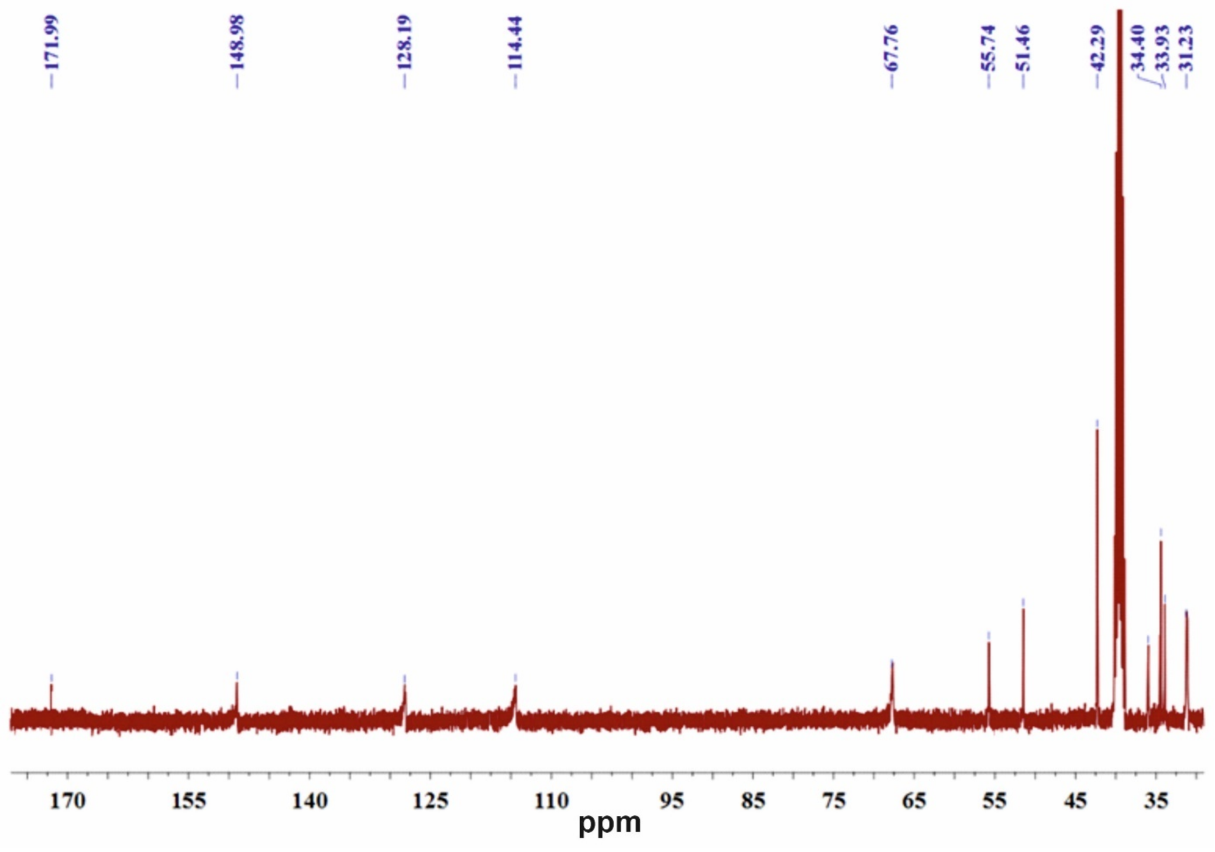

Figure S8. ^{13}C NMR spectrum of 4,8,14,18,23,26,28,31,32,35-deca-((2-(morfolin)ethylcarbamoyl-2-sulfanedil)ethoxy)-pillar[5]arene **(6)**. DMSO-d_6, 298 K, 400 MHz.

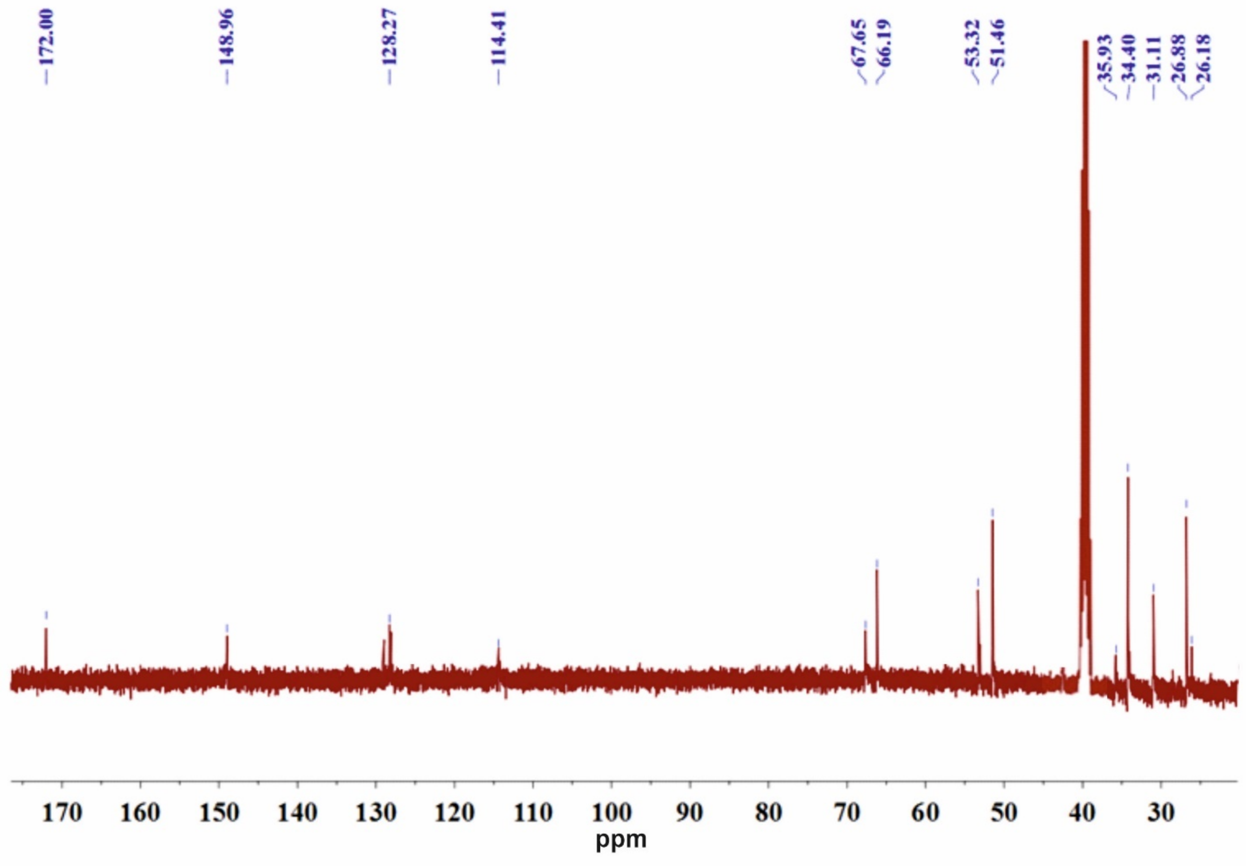

Figure S9. Mass spectrum (MALDI-TOF, 4-nitroaniline matrix) of 4,8,14,18,23,26,28,31,32,35–deca-((hydrazidocarbonil-2-sulfanedyl)ethoxy)- pillar[5]arene **(3)**.

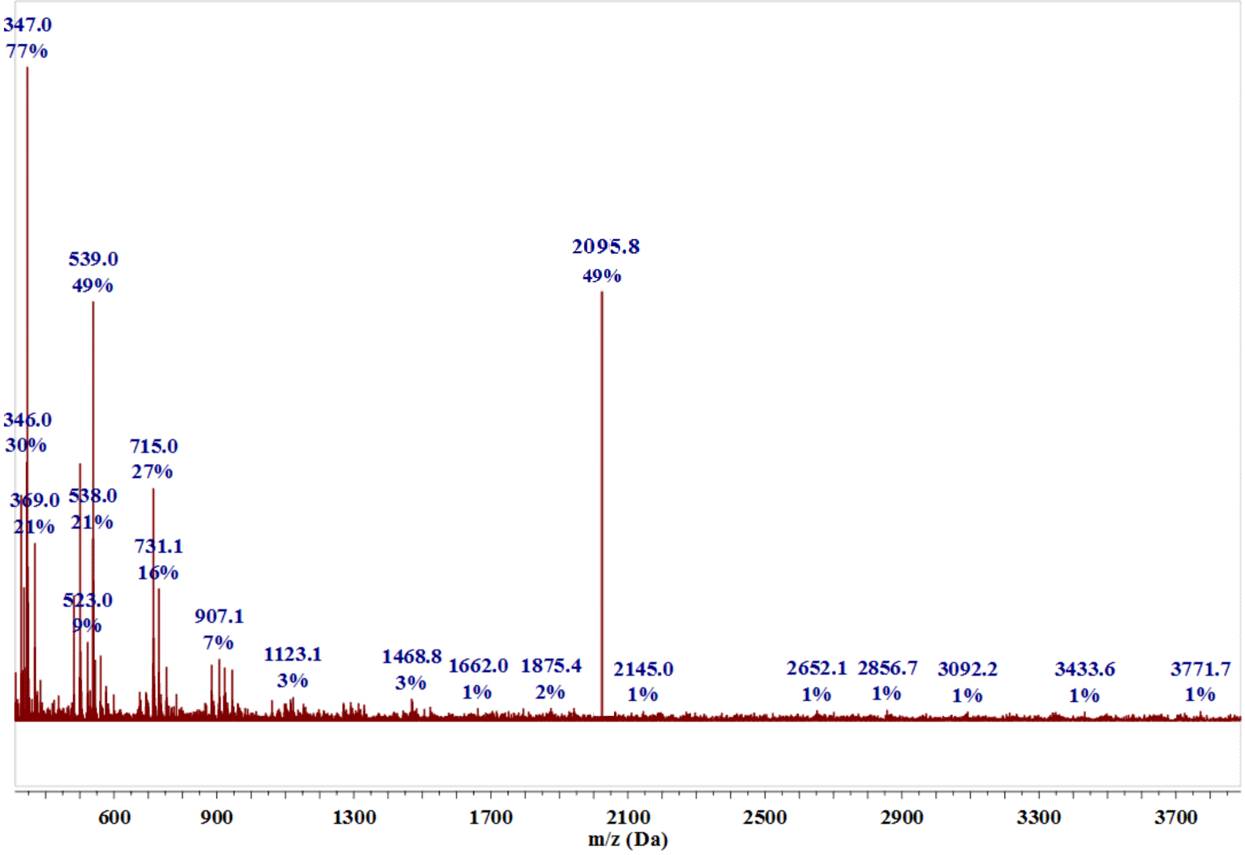

Figure S10. Mass spectrum (MALDI-TOF, 4-nitroaniline matrix) of 4,8,14,18,23,26,28,31,32,35–deca-((2-(N,N-diethyl)ethylcarbamoyl-2-sulfanedil)ethoxy)-pillar[5]arene **(4)**.

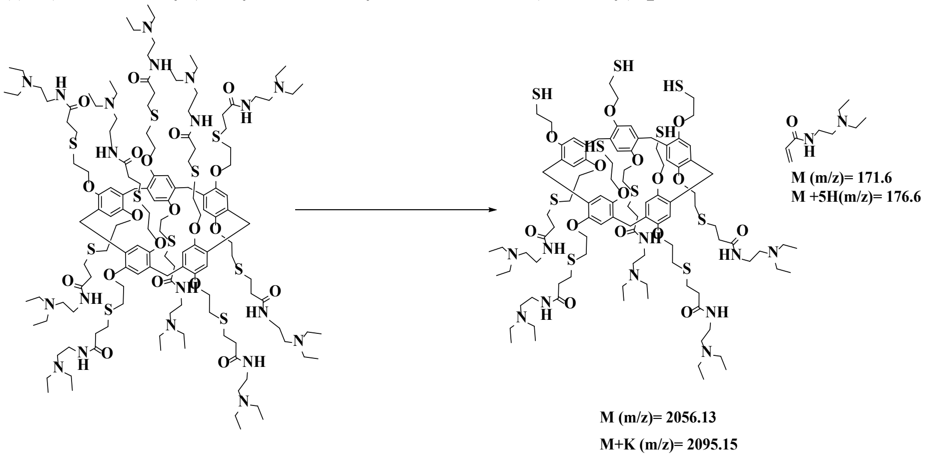

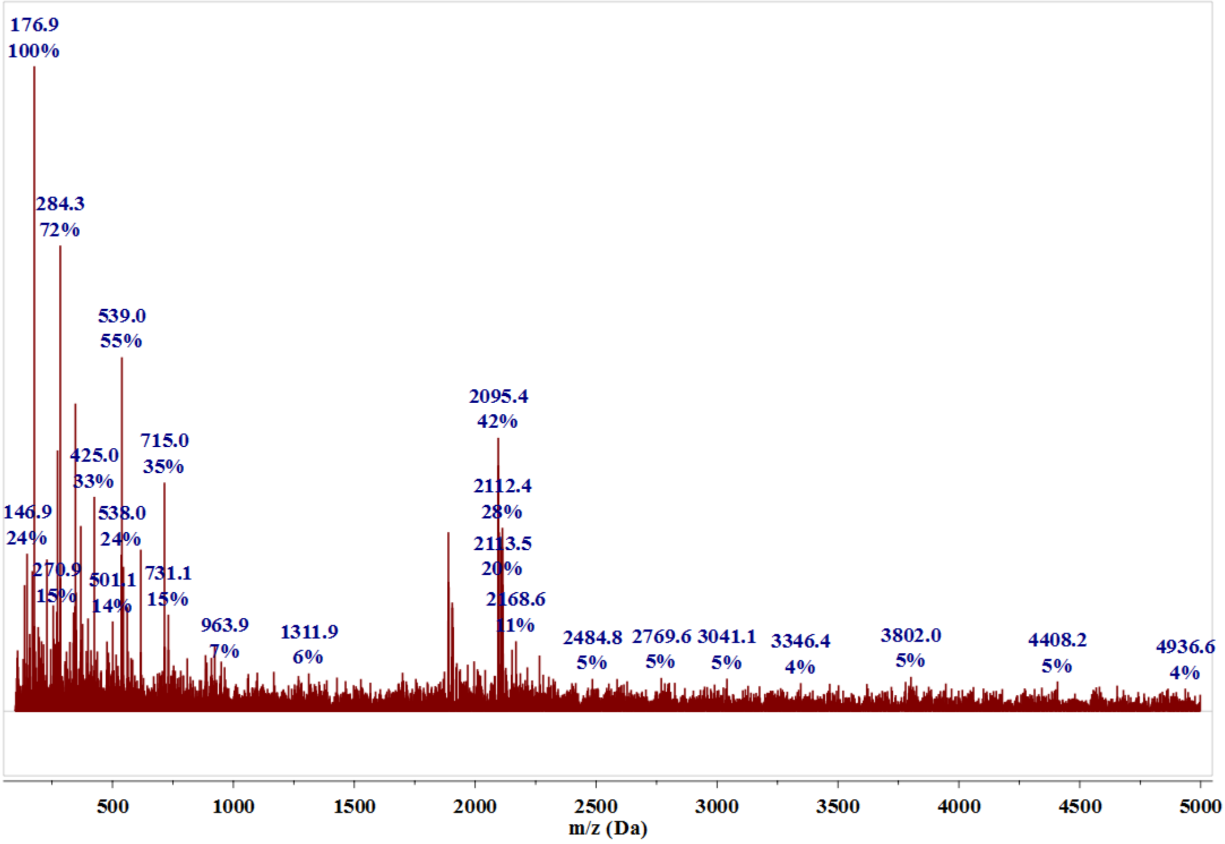

Figure S11. Mass spectrum (MALDI-TOF, 4-nitroaniline matrix) of 4,8,14,18,23,26,28,31,32,35-deca-((2-(N,N-dimethyl)ethylcarbamoyl-2-sulfanedil)ethoxy)-pillar[5]arene **(5)**.

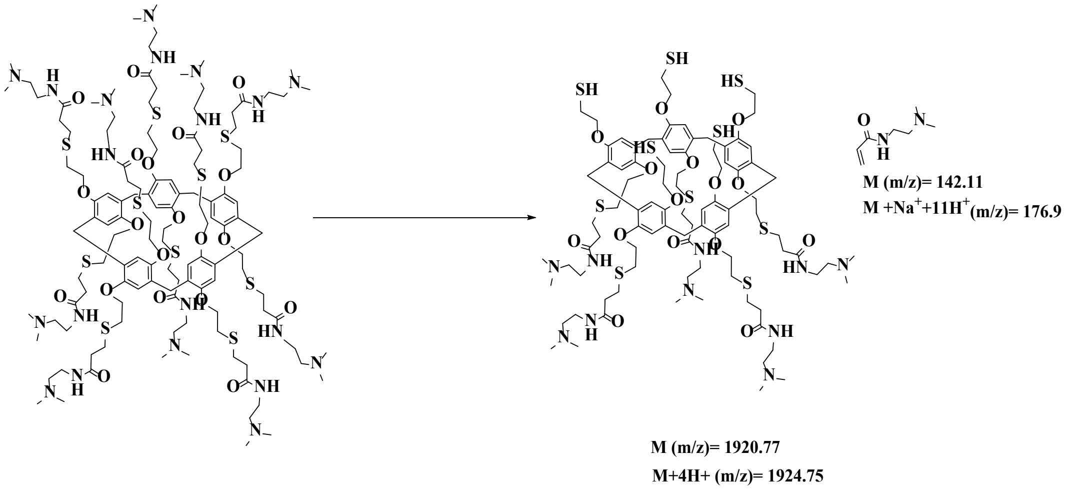

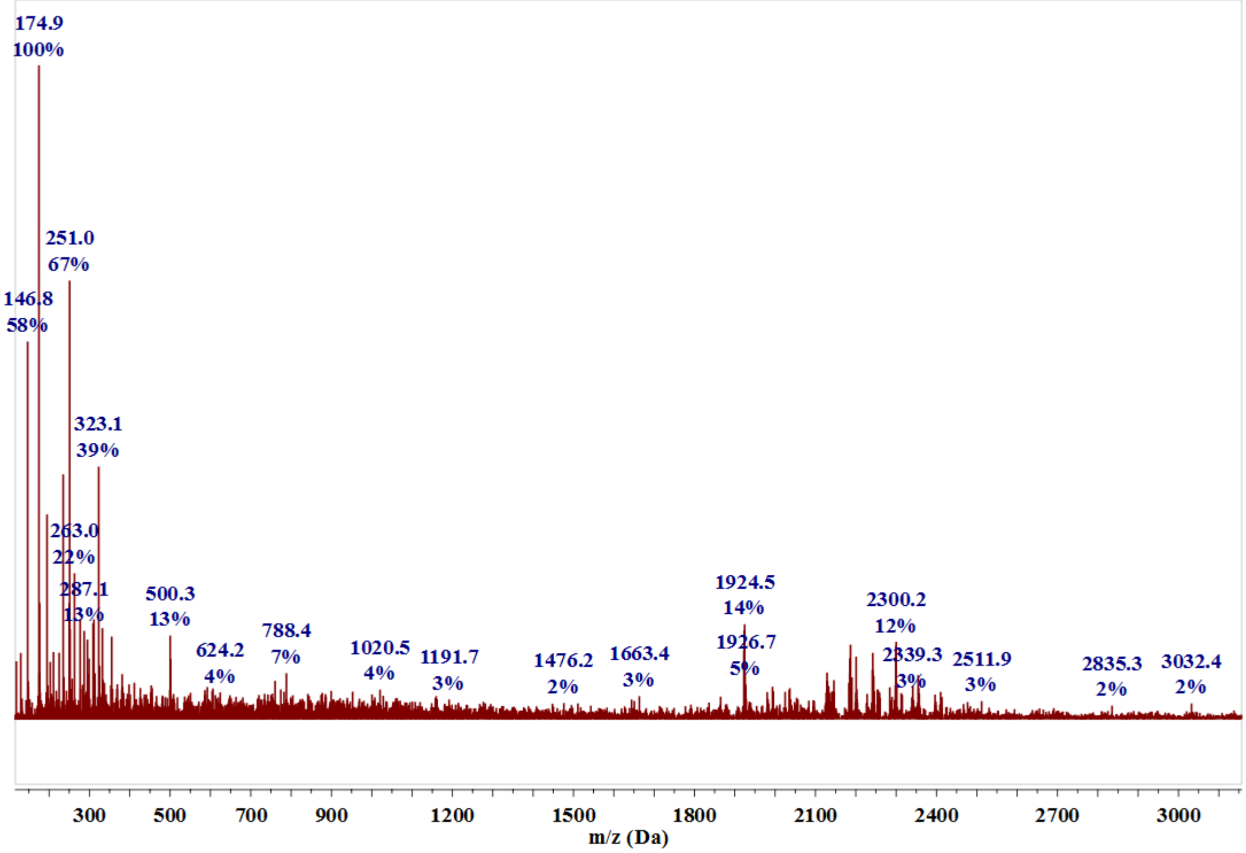

Figure S12. Mass spectrum (MALDI-TOF, 4-nitroaniline matrix) of 4,8,14,18,23,26,28,31,32,35-deca-((2-(morfolin)ethylcarbamoyl-2-sulfanedil)ethoxy)-pillar[5]arene **(6)**.

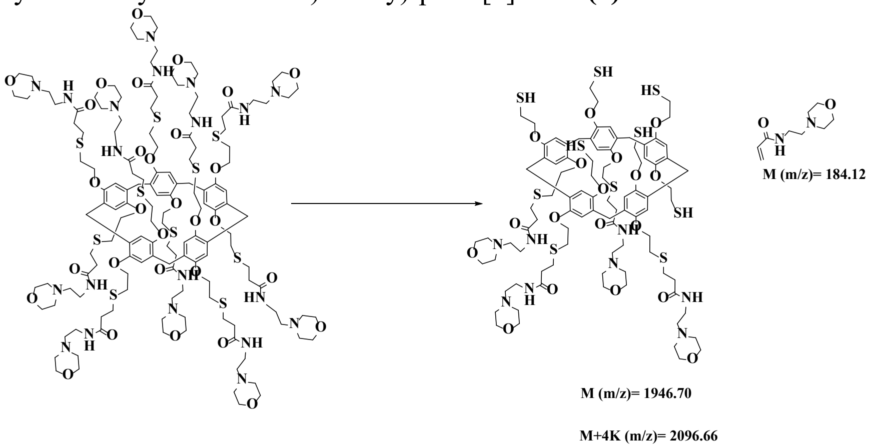

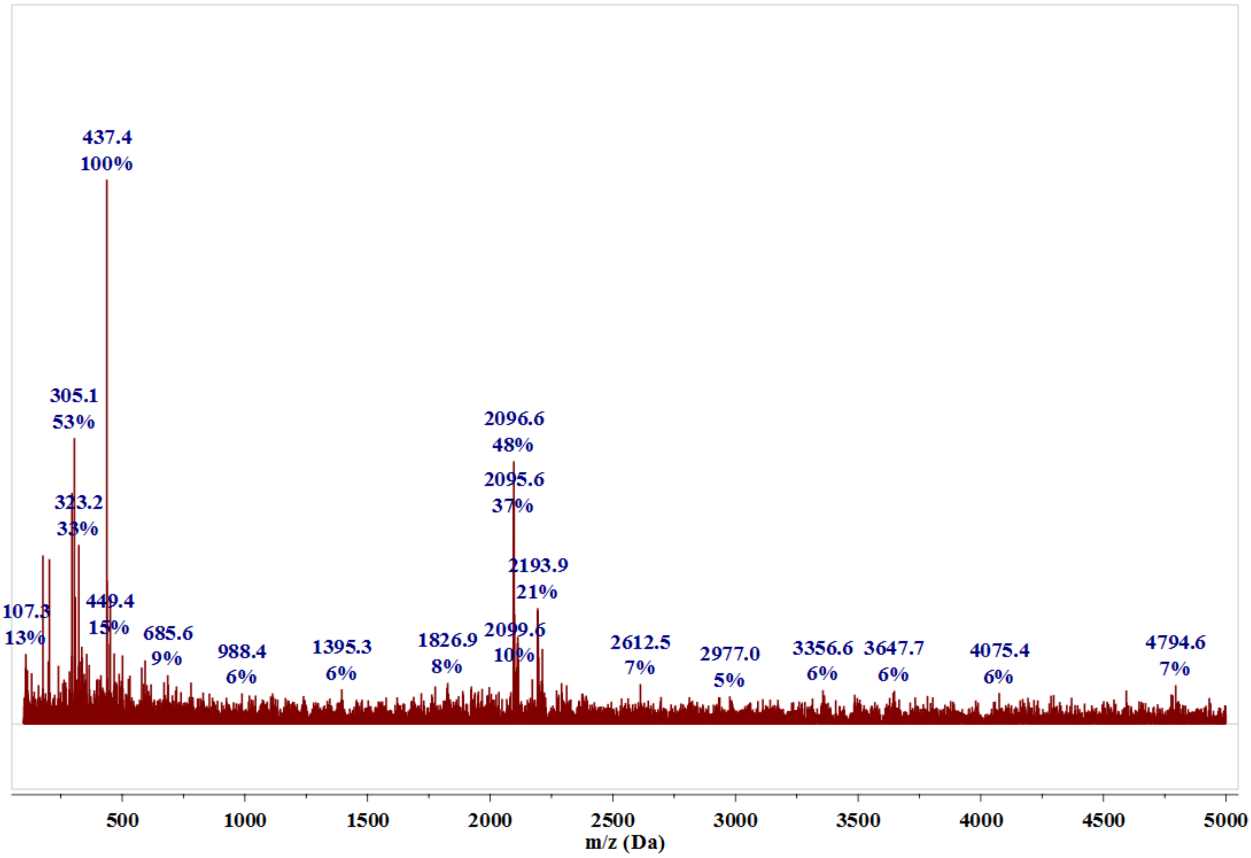

Figure S13. IR spectrum of 4,8,14,18,23,26,28,31,32,35–deca-((hydrazidocarbonil-2-sulfanedyl)ethoxy)-pillar[5]arene **(3)**.

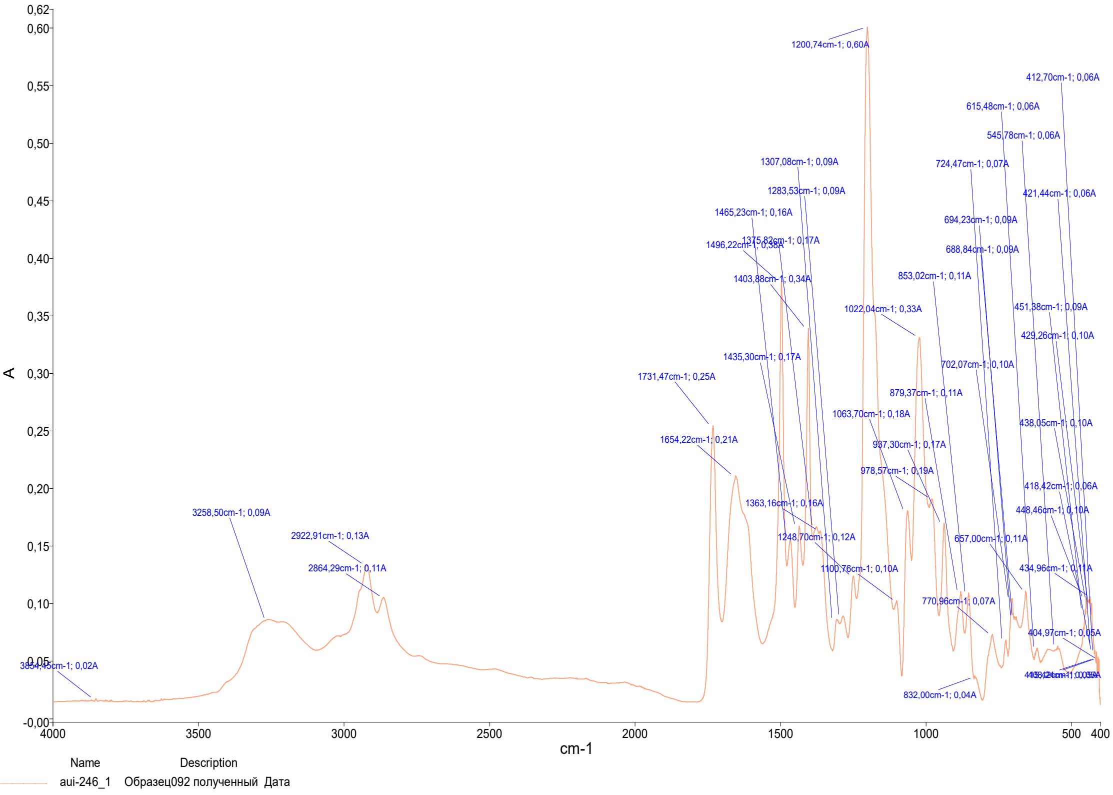

Figure S14. IR spectrum of 4,8,14,18,23,26,28,31,32,35–deca-((2-(N,N-diethyl)ethylcarbamoyl-2-sulfanedil)ethoxy)-pillar[5]arene **(4)**.

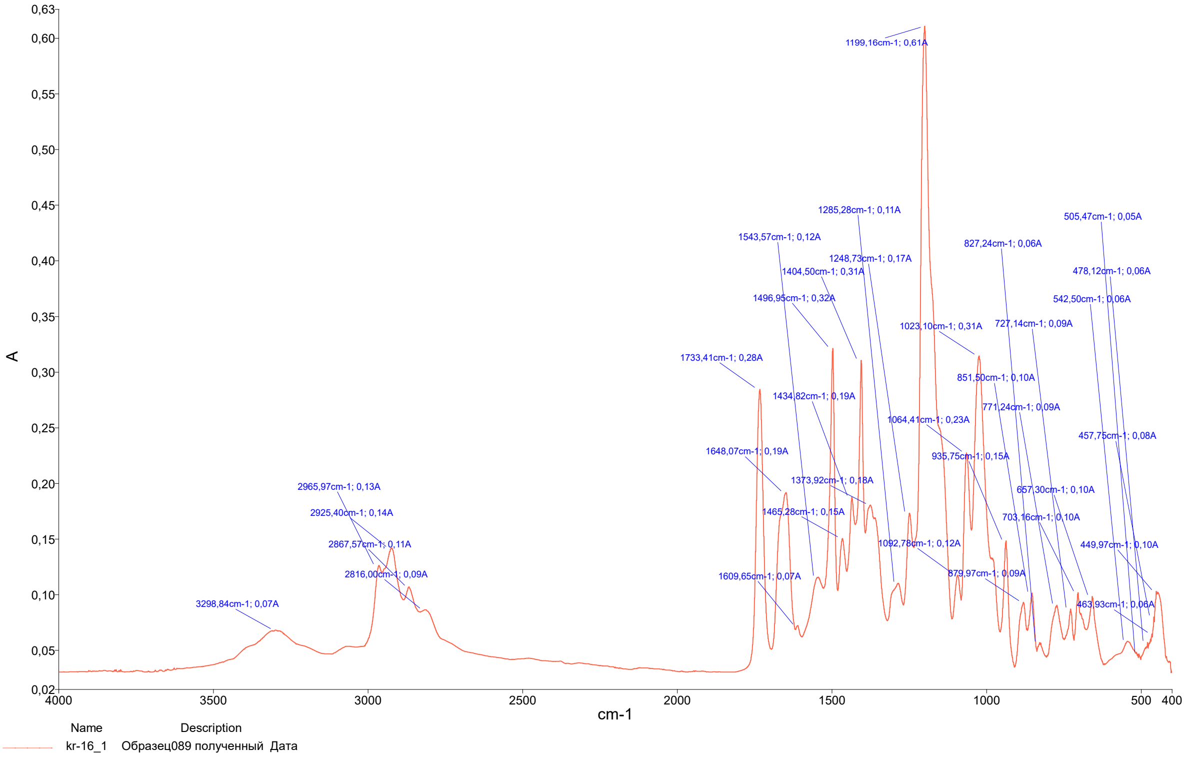

Figure S15. IR spectrum of 4,8,14,18,23,26,28,31,32,35-deca-((2-(N,N-dimethyl)ethylcarbamoyl-2-sulfanedil)ethoxy)-pillar[5]arene **(5)**.

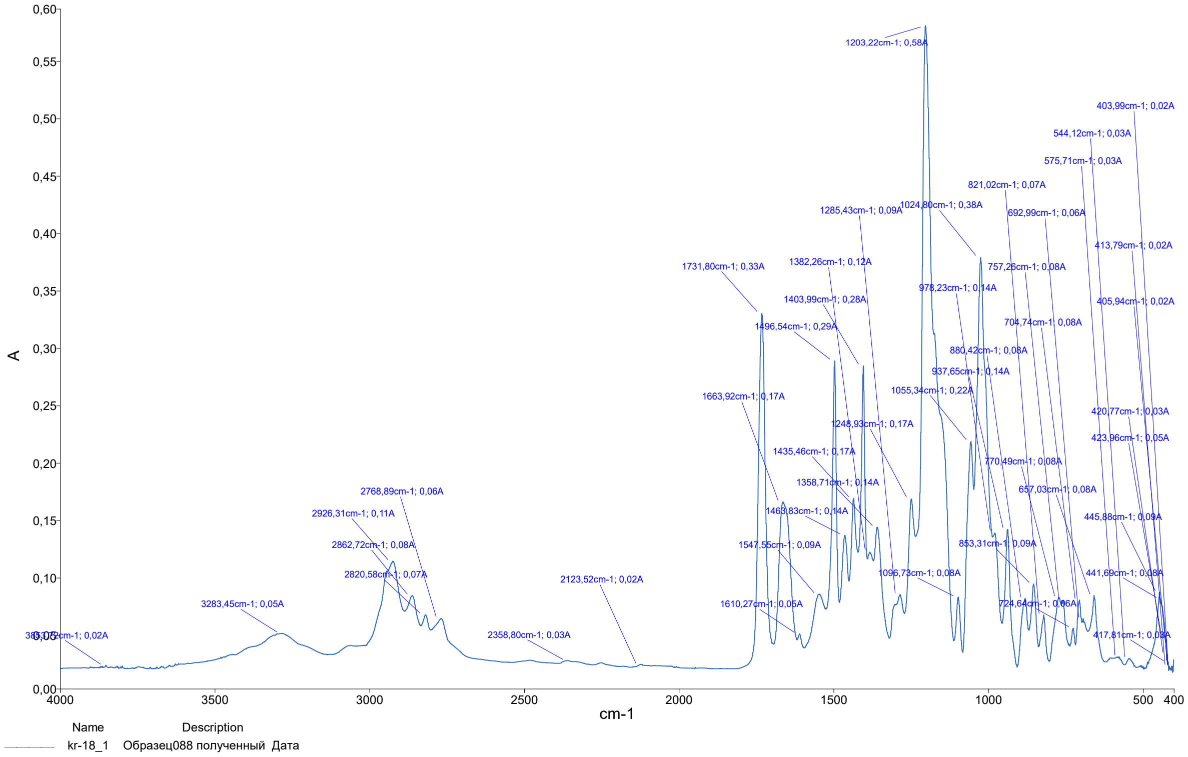

Figure S16. IR spectrum of 4,8,14,18,23,26,28,31,32,35-deca-((2-(morfolin)ethylcarbamoyl-2-sulfanedil)ethoxy)-pillar[5]arene **(6)**.

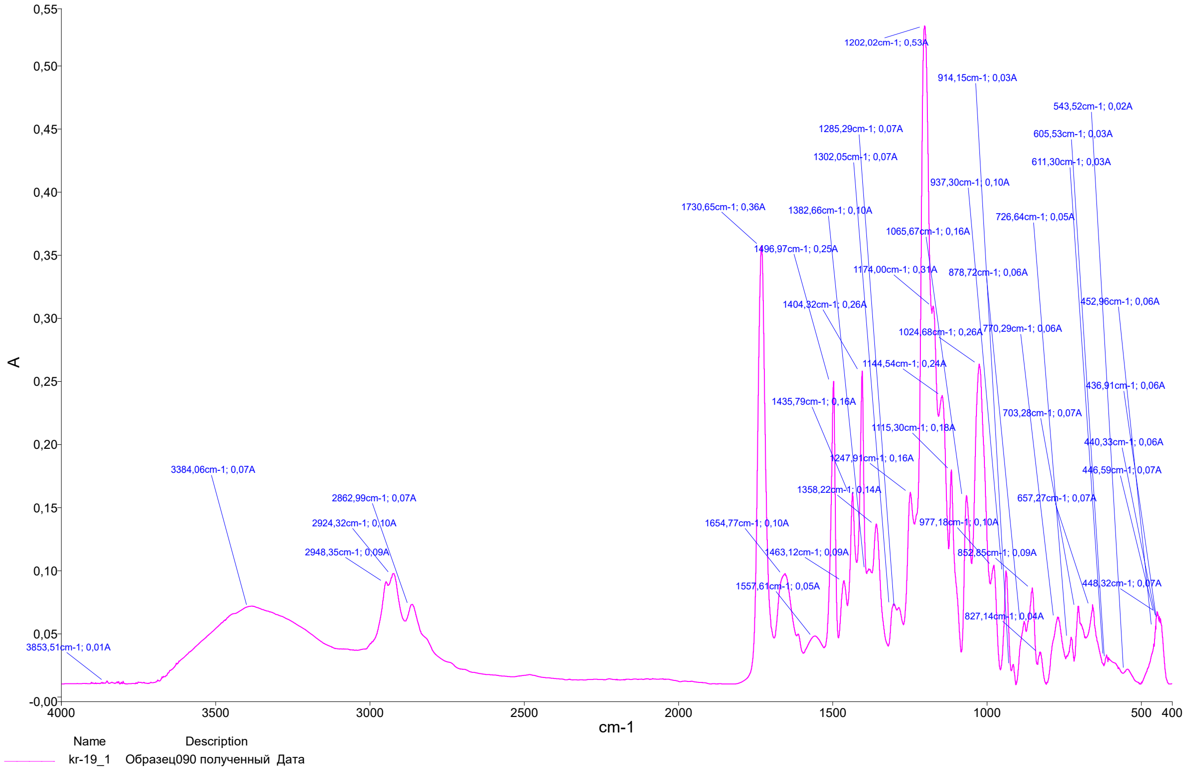

3. Dynamic light scattering

Table S1. Aggregation **4-6** with DNA $_{\text{salmon sperm}}$ in 50 mM Tris-HCl buffer (pH =6.5) by DLS method.

Sample	C $_{\text{Host}}$, M	C $_{\text{DNA base pairs}}$, M	PDI	d $_{\text{average}}$, nm	ζ-potential, мВ
**4**	5×10^{-5}	0	**0.092±0.029**	**348±2**	
	1×10^{-5}	0	0.21±0.02	369±3	
	1×10^{-6}	0	0.57±0.05	721±57	
**5**	5×10^{-5}	0	0.45±0.06	449±21	
	1×10^{-5}	0	0.61±0.04	1401±73	
	1×10^{-6}	0	0.57±0.09	1562±70	
**6**	5×10^{-5}	0	0.38±0.05	909±45	
	1×10^{-5}	0	0.53±0.06	1248±46	
	1×10^{-6}	0	0.59±0.12	1470±69	
**DNA** $_{\text{salmon sperm}}$	0	6×10^{-4}	0.79±0.05	803±86	
	0	6×10^{-5}	0.62±0.08	1444±61	
	0	3×10^{-4}	0.59±0.04	1311±113	
**4/DNA** $_{\text{salmon sperm}}$	1×10^{-5}	3×10^{-4}	**0.24±0.02**	**220±10**	**19.3±0.7**
**5/DNA** $_{\text{salmon sperm}}$	1×10^{-5}	3×10^{-4}	**0.28±0.07**	**114±4**	**20±0.8**
**6/DNA** $_{\text{salmon sperm}}$	1×10^{-5}	3×10^{-4}	0.42±0.04	345±25	19.8±0.5

Figure S17. Size distribution of the particles by intensity for **4** 10^{-4} M in 50 mM Tris-HCl buffer (pH =6.5) (PDI = 0.092±0.029, d_{av} = 348±2 nm).

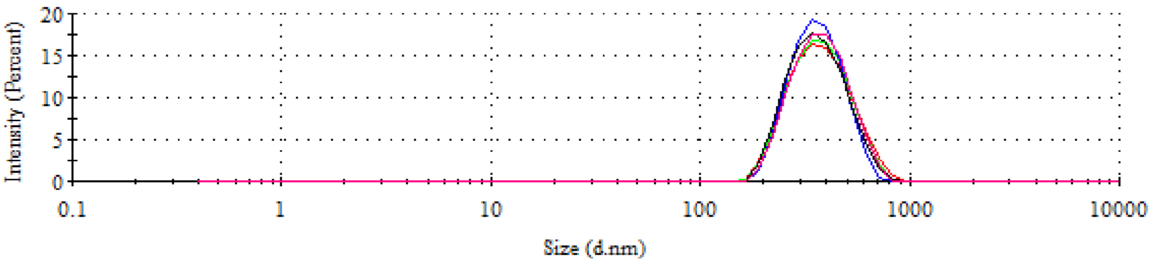

Figure S18. Size distribution of the particles by intensity for **5** 10^{-4} M in 50 mM Tris-HCl buffer (pH =6.5) (PDI = 0.45±0.06, d_{av} = 449±21 nm).

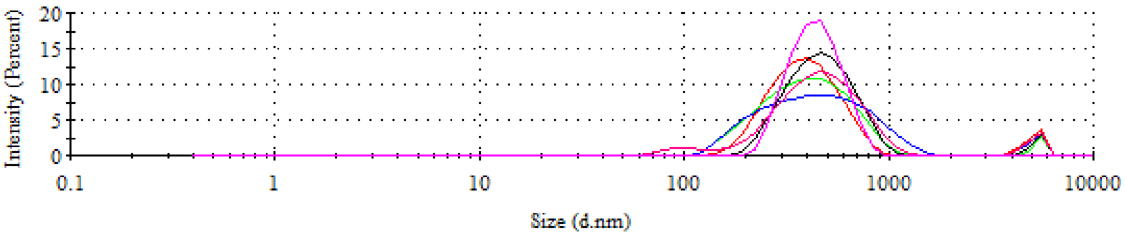

Figure S19. Size distribution of the particles by intensity for **6** 10^{-4} M in 50 mM Tris-HCl buffer (pH =6.5) (PDI = 0.38±0.05, d_{av} = 909±45 nm).

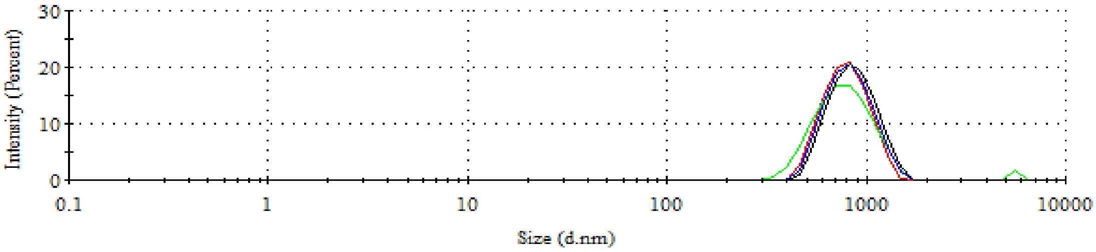

Figure S20. Size distribution of the particles by intensity for **DNA** **salmon sperm** 6×10^{-5} M base pairs in 50 mM Tris-HCl buffer (pH =6.5) (PDI = 0.092±0.029, d_{av} = 348±2 nm).

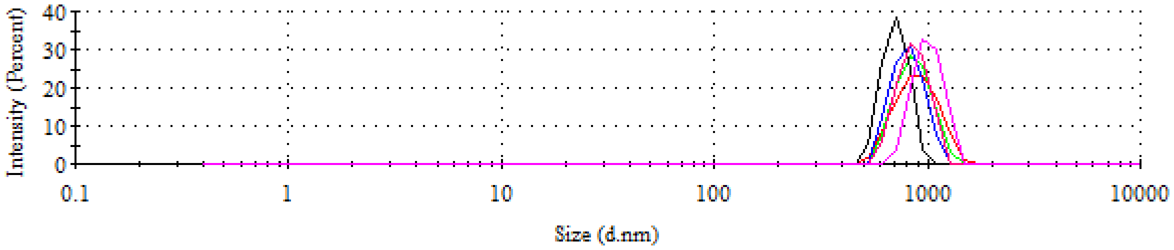

Figure S21. Size distribution of the particles by intensity for **4** 10^{-5} M + **DNA** **salmon sperm** 6×10^{-5} M base pairs in 50 mM Tris-HCl buffer (pH =6.5) (PDI = 0.24±0.02, d_{av} = 220±10 nm).

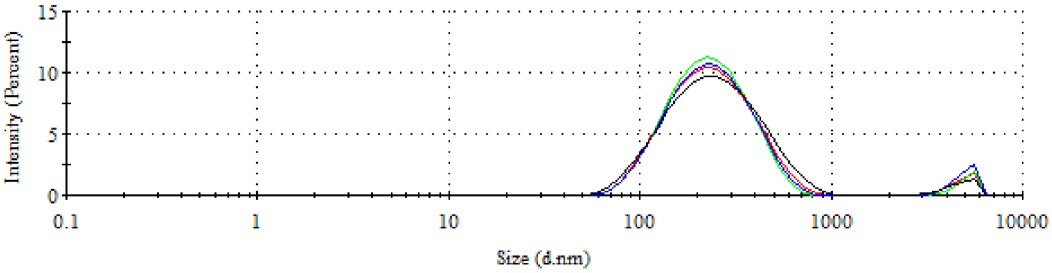

Figure S22. Size distribution of the particles by intensity for **5** 10^{-5} M + **DNA** **salmon sperm** 6×10^{-5} M base pairs in 50 mM Tris-HCl buffer (pH =6.5) (PDI = 0.28±0.07, d_{av} = 114±4 nm).

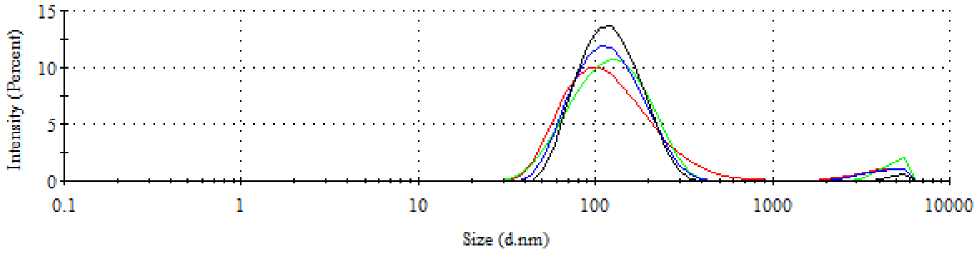

Figure S23. Size distribution of the particles by intensity for **6** 10^{-5} M + **DNA** **salmon sperm** 6×10^{-5} M base pairs in 50 mM Tris-HCl buffer (pH =6.5) (PDI = 0.42±0.04, d_{av} = 345±25 nm).

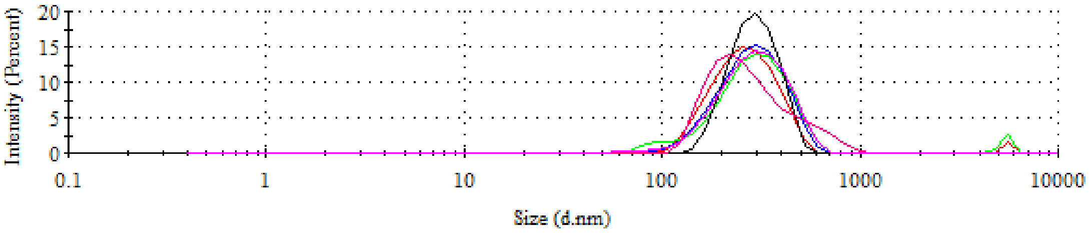

4. UV-vis study

Figure S24. Absorption spectra of macrocycle **4** (1×10^{-5} M), DNA $_{\text{salmon sperm}}$ (6×10^{-5} M base pairs), macrocycle **3** (1×10^{-5} M) with DNA $_{\text{salmon sperm}}$ (6×10^{-5} M base pairs) in 50 mM Tris-HCl buffer (pH =6.5).

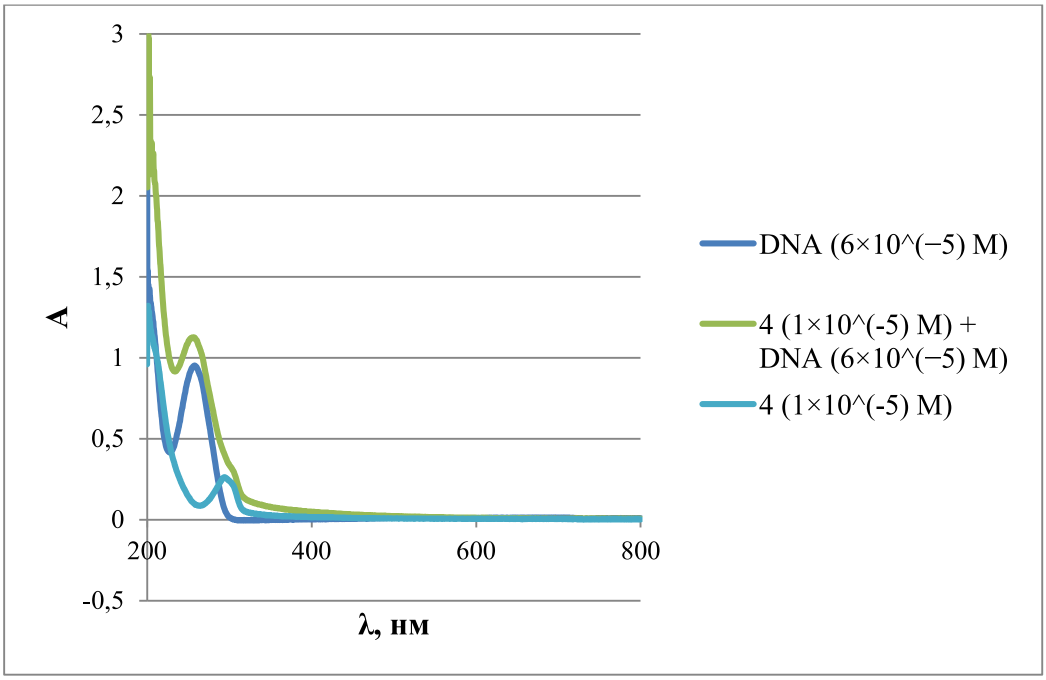

Figure S25. Absorption spectra of macrocycle **5** (1×10^{-5} M), DNA $_{\text{salmon sperm}}$ (6×10^{-5} M base pairs), macrocycle **5** (1×10^{-5} M) with DNA $_{\text{salmon sperm}}$ (6×10^{-5} M base pairs) in 50 mM Tris-HCl buffer (pH =6.5).

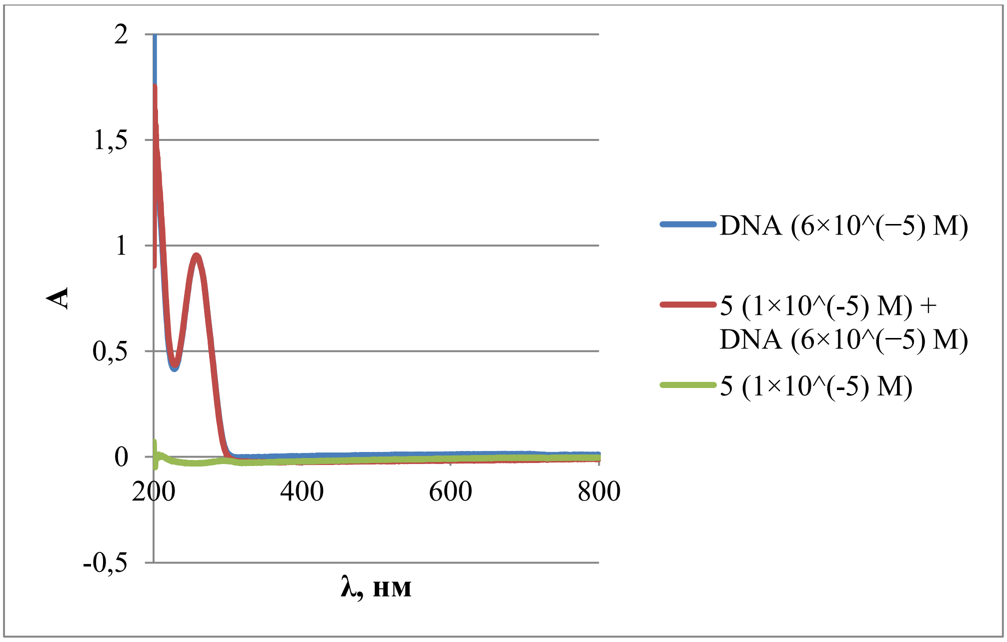

Figure S26. Absorption spectra of macrocycle **6** (1×10^{-5} M), DNA $_{\text{salmon sperm}}$ (6×10^{-5} M base pairs), macrocycle **6** (1×10^{-5} M) with DNA $_{\text{salmon sperm}}$ (6×10^{-5} M base pairs) in 50 mM Tris-HCl buffer (pH =6.5).

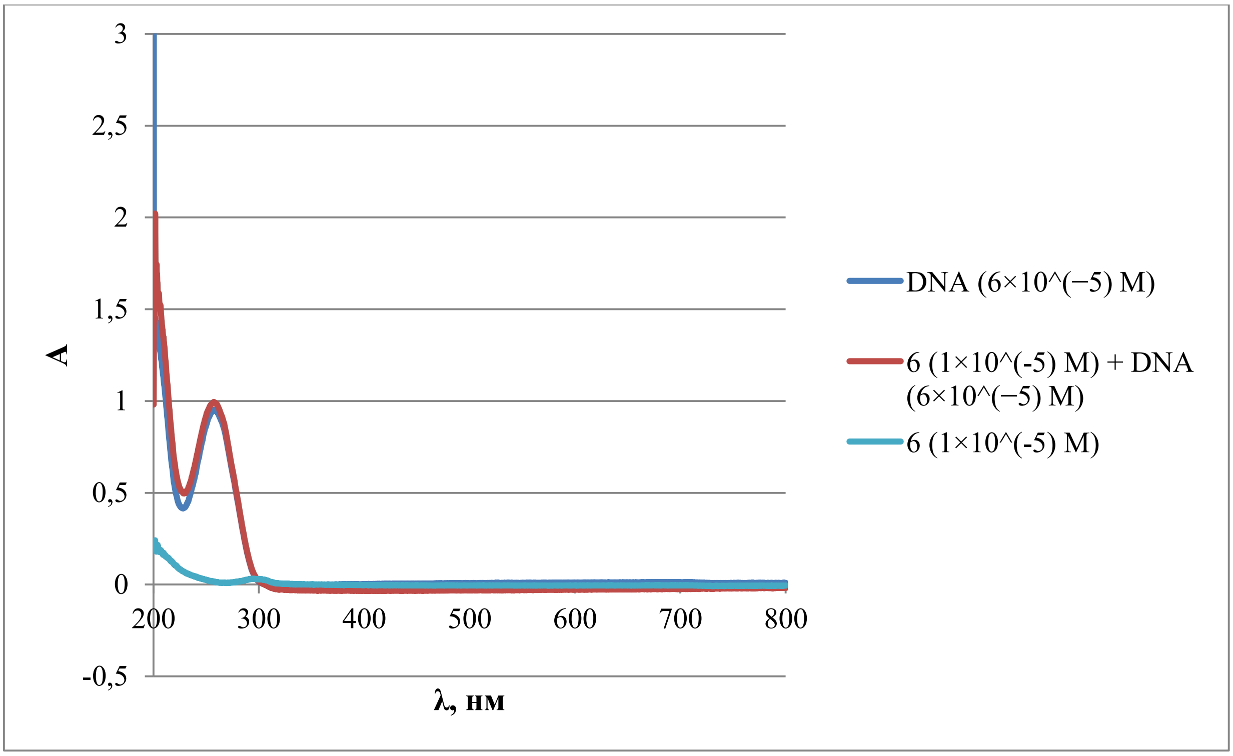

Figure S27. Absorption spectra of the system macrocycle **4** (1×10^{-5} M)/ DNA $_{\text{salmon sperm}}$ (2×10^{-5}-3×10^{-4} M base pairs) in 50 mM Tris-HCl buffer (pH =6.5).

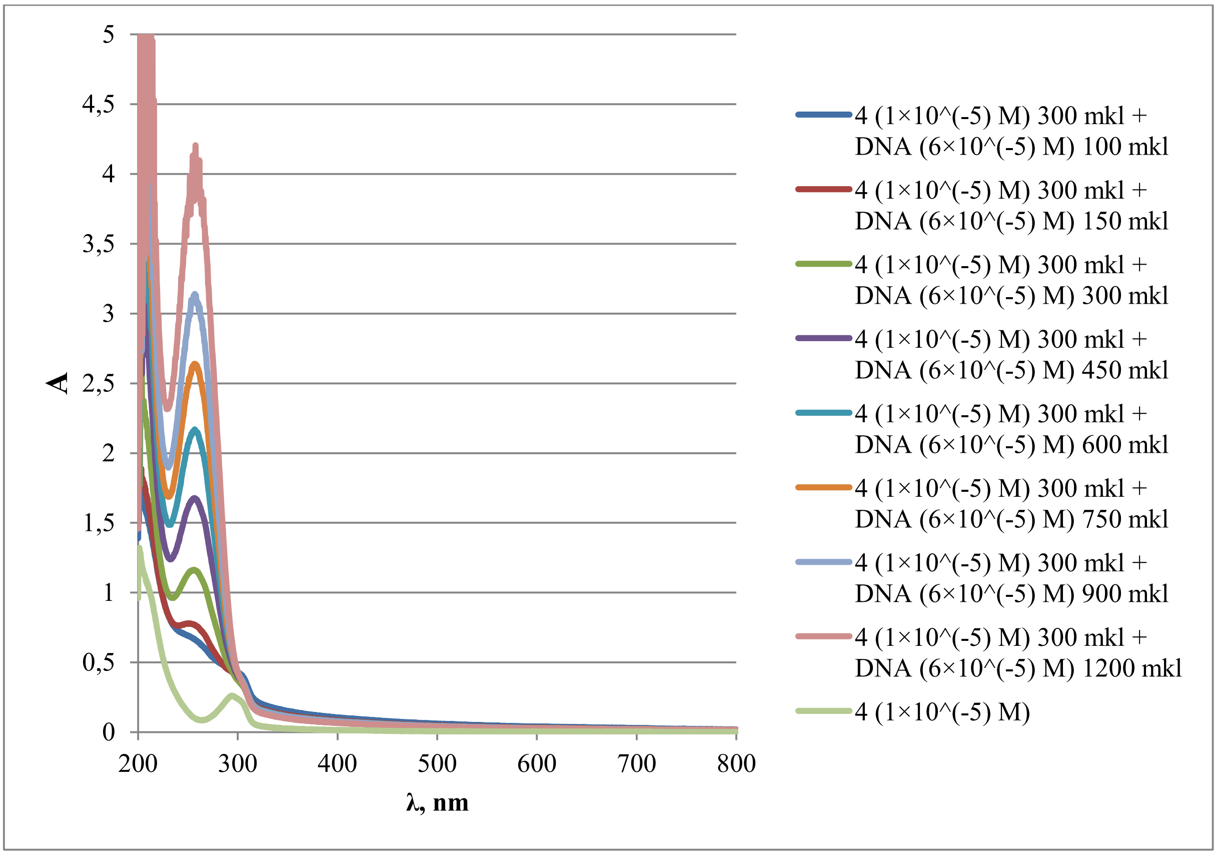

Figure S28. Absorption spectra of the DNA $_{salmon\ sperm}$ (2×10^{-5}-3×10^{-4} M base pairs) in 50 mM Tris-HCl buffer (pH =6.5).

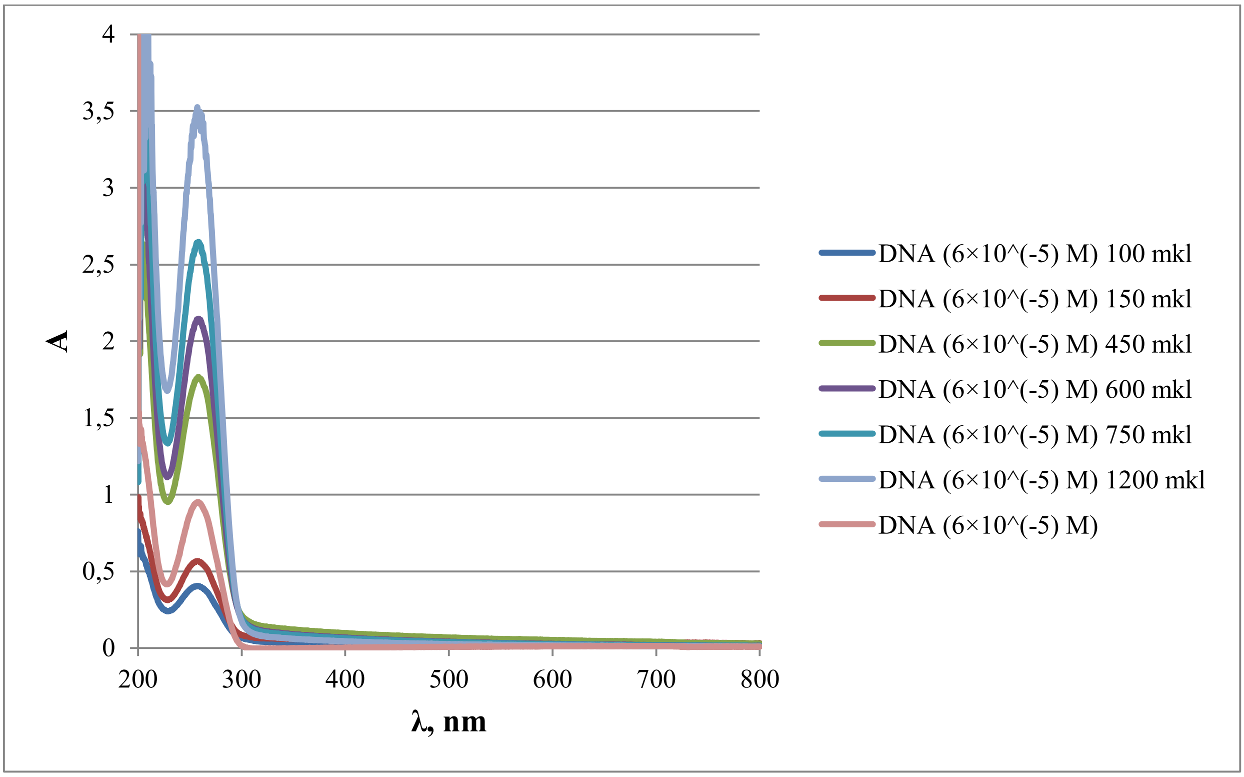

Table S2. Interaction of macrocycles **4-6** with excess metal cations by UV-*vis* study.

<table>
<tr><th rowspan="2"></th><th colspan="2">Ag^{+}</th><th colspan="2">Cu^{2+}</th><th colspan="2">Fe^{3+}</th><th colspan="2">Ni^{2+}</th><th colspan="2">Co^{2+}</th><th colspan="2">Cd^{2+}</th><th colspan="2">Cu^{+}</th></tr>
<tr><th>ΣA</th><th>A complex</th><th>ΣA</th><th>A complex</th><th>ΣA</th><th>A complex</th><th>ΣA</th><th>A complex</th><th>ΣA</th><th>A complex</th><th>ΣA</th><th>A complex</th><th>ΣA</th><th>A complex</th></tr>
<tr><td rowspan="2">**4** CH_3OH λ=294 nm A=0.273</td><td rowspan="2">0.308</td><td rowspan="2">0.281</td><td rowspan="2">0.347</td><td rowspan="2">0.387</td><td>0.304 λ=360</td><td>0.302</td><td rowspan="2">0.273</td><td rowspan="2">0.301</td><td rowspan="2">0.289</td><td rowspan="2">0.300</td><td rowspan="2">0.273</td><td rowspan="2">0.309</td><td rowspan="2"></td><td rowspan="2"></td></tr>
<tr><td>0.472 λ=294</td><td>0.506</td></tr>
<tr><td rowspan="2">**5** CH_3OH λ=294 nm A=0.266</td><td rowspan="2">0.301</td><td rowspan="2">0.253</td><td rowspan="2">0.340</td><td rowspan="2">0.387</td><td>0.304 λ=360</td><td>0.344</td><td rowspan="2">0.266</td><td rowspan="2">0.286</td><td rowspan="2">0.282</td><td rowspan="2">0.295</td><td rowspan="2">0.266</td><td rowspan="2">0.264</td><td rowspan="2"></td><td rowspan="2"></td></tr>
<tr><td>0.465 λ=294</td><td>0.481</td></tr>
<tr><td rowspan="2">**6** CH_3CN λ=295 nm A=0.323</td><td rowspan="2">0.323</td><td rowspan="2">0.284</td><td rowspan="2">0.402</td><td rowspan="2">0.359</td><td rowspan="2">0.323</td><td rowspan="2">0.291</td><td rowspan="2">0.323</td><td rowspan="2">0.264</td><td rowspan="2">0.323</td><td rowspan="2">0.303</td><td rowspan="2">0.323</td><td rowspan="2">0.294</td><td>0.323 λ=294</td><td>0.291</td></tr>
<tr><td>1.594 λ=243</td><td>1.594</td></tr>
</table>

Figure S29. Bindfit (Fit data to 1:1, 1:2 and 2:1 Host-Guest equilibria) screenshots taken from the summary window of the website supramolecular.org. These screenshots shows the raw data for UV-*vis* titration of **4** with DNA $_{\text{salmon sperm}}$, the data fitted to 1:1 binding model (a), 1:2binding model (b) and 2:1 binding model (c).

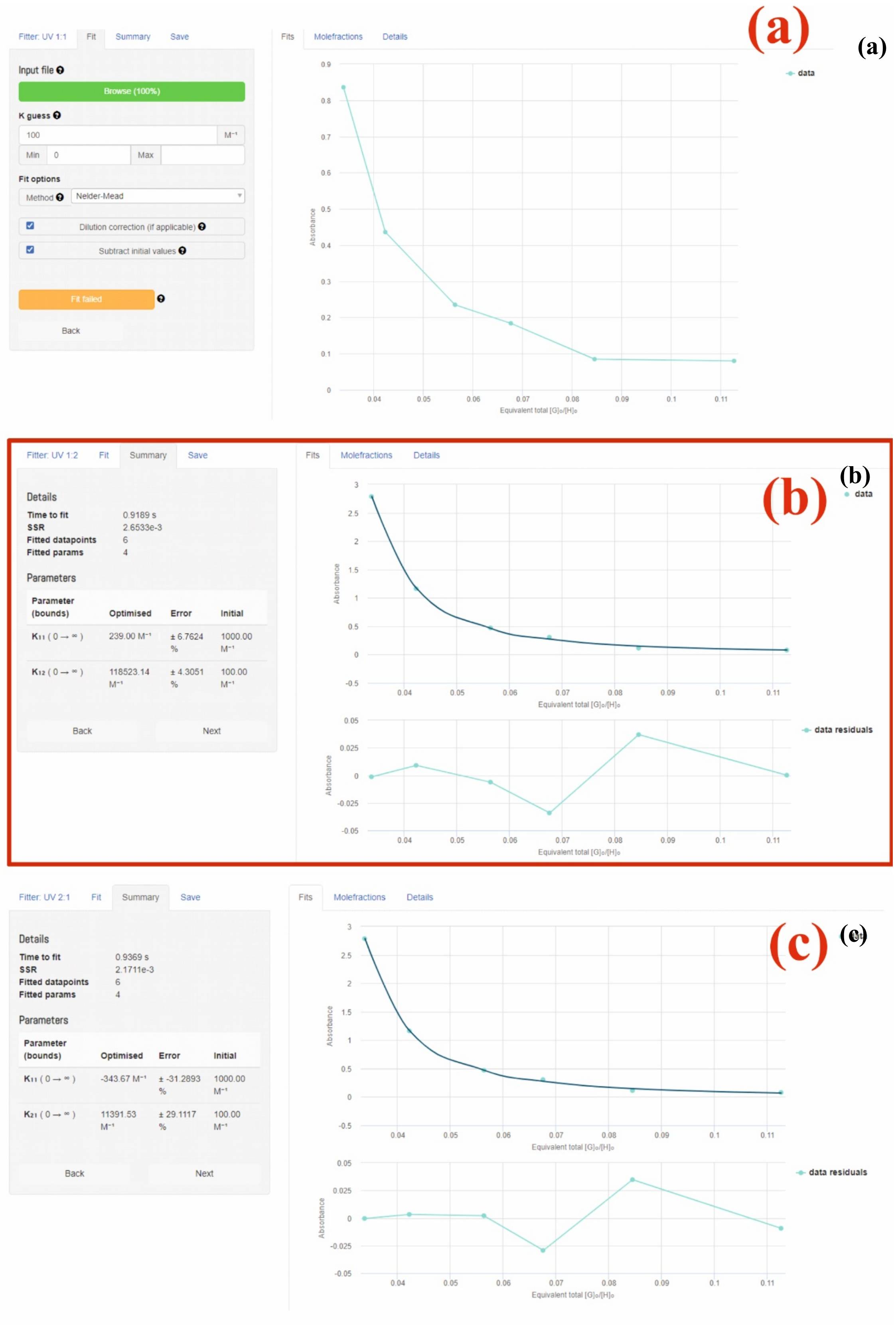

5. Biological research

Table S3. Light absorption of eluate after 48 hours of incubation of *S. aureus* culture in the presence of different concentrations of pillar[5]arene **4**

Repetition of the experiment / Concentration of **4**, M	1	2	3	4
1×10^{-3}	0.402	0.306	0.447	
1×10^{-4}	0.252	0.311	0.38	0.288
1×10^{-5}	0.239	0.314	0.252	0.24
1×10^{-6}	0.298	0.261	0.328*	0.258
Control	0.231	0.29	0.304	0.325

* The biofilm in one of the replicates at a pillararene concentration of 1×10^{-6} M was damaged when washed with distilled water. The results of this cell were not taken into account in the analysis (the data of the cells are marked * in Table S3)

8. References

S1. Yao, Y.; Xue, M.; Chi, X.; Ma, Y.; He, J.; Abliz, Z.; Huang, F. A new water-soluble pillar[5]arene: synthesis and application in the preparation of gold nanoparticles. *Chem. Commun.* **2012,** *48*, 6505. https://doi.org/10.1039/c2cc31962d

S2. Shurpik, D.N.; Mostovaya, O.A.; Sevastyanov, D.A.; Lenina, O.A.; Sapunova, A.S.; Voloshina, A.D.; Petrov, K.A.; Kovyazina, I.V.; Cragg, P.J.; Stoikov, I.I. Supramolecular neuromuscular blocker inhibition by a pillar[5]arene through aqueous inclusion of rocuronium bromide. *Org. amp; Biomol. Chem.* **2019,** *17*, 9951-9959. https://doi.org/10.1039/c9ob02215e

S3. Mosmann, T. Rapid colorimetric assay for cellular growth and survival: application to proliferation and cytotoxicity assays. *J. Immunol. Methods.* **1983,** *65*, 55–63. https://doi.org/10.1016/0022-1759(83)90303-4
